# The Association between Sarcoidosis and Ischemic Heart Disease—A Healthcare Analysis of a Large Israeli Population

**DOI:** 10.3390/jcm10215067

**Published:** 2021-10-29

**Authors:** Tal Gonen, Daphna Katz-Talmor, Howard Amital, Doron Comaneshter, Arnon D. Cohen, Shmuel Tiosano

**Affiliations:** 1 Sackler Faculty of Medicine, Tel-Aviv University, Tel-Aviv 6997801, Israel; tal.2279@gmail.com (T.G.); Howard.Amital@sheba.health.gov.il (H.A.); 2 Sheba Medical Center, Department of Medicine ‘B’, Tel-Hashomer, Ramat-Gan 5266202, Israel; 3 Department of Medicine ‘A’, University Hospital Samason Assuta Ashdod, Ashdod 7747629, Israel; Katzdaphna@gmail.com; 4 Zabludowicz Center for Autoimmune Diseases, Sheba Medical Center, Tel-Hashomer, Ramat-Gan 5266202, Israel; 5 Chief Physician’s Office, Clalit Health Services Tel Aviv, Tel-Aviv 6209813, Israel; doronko1@clalit.org.il (D.C.); arcohen@clalit.org.il (A.D.C.); 6 Faculty of Health Sciences, Siaal Research Center for Family Medicine and Primary Care, Ben Gurion University of the Negev, Beer Sheva 8410501, Israel; 7 The Leviev Heart Center, Sheba Medical Center, Tel-Hashomer, Ramat-Gan 5266202, Israel

**Keywords:** sarcoidosis, ischemic heart disease, comorbidities

## Abstract

(1) Background: Inflammation plays a pivotal role in atherosclerosis, and the association between chronic inflammatory states and ischemic heart disease (IHD) has been shown in several rheumatic diseases. Persistent inflammation might also be a risk factor for IHD in sarcoidosis patients. (2) Methods: Demographic and clinical data of 3750 sarcoidosis patients and 18,139 age- and sex-matched controls were retrieved from the database of Clalit Health Services, Israel’s largest healthcare organization. Variables associated with IHD were assessed by a logistic regression model. To assess for variables that were related to increased risk of all-cause mortality, the Cox proportional hazards method was used, and a log-rank test was performed for survival analysis. (3) Results: Both groups were composed of 64% females with a median age of 56 years. An association between sarcoidosis and IHD was demonstrated by a multivariate analysis (adjusted odds ratio (OR) 1.5; 95% confidence interval (CI) 1.36–1.66). Long-term follow-up revealed increased mortality among sarcoidosis patients: 561 (15%) deaths compared to 1636 (9%) deaths among controls (*p* < 0.001). Survival analysis demonstrated that sarcoidosis patients were also at increased risk for all-cause mortality compared to controls (multivariate model, adjusted HR 1.93; 95% CI 1.76–2.13).

## 1. Introduction

Ischemic heart disease (IHD) is considered a leading cause of premature mortality worldwide [1,2]. Potential etiologies of IHD include occlusion of the coronary arteries by plaque, coronary artery spasm, and coronary microvascular dysfunction [3]. Research over the past two decades suggested that inflammation plays a role in atherosclerosis [4]. Atherosclerosis was shown to be associated with various inflammatory states, ranging from local inflammation (e.g., periodontitis), to systemic rheumatic diseases, such as rheumatoid arthritis and systemic lupus erythematosus [5,6,7]. Sarcoidosis is an inflammatory disease characterized pathologically by the formation of non-caseating granulomas [8]. The clinical presentation of sarcoidosis varies greatly with respect to the affected organ or system. Common manifestations of the disease can be categorized as either pulmonary or extra-pulmonary disease, the latter most commonly involving the eyes, skin, and liver. Fifty percent of patients experience spontaneous disease resolution within 2 years of diagnosis, and relapse in patients who experienced spontaneous remission is considered to be rare [9]. One study conducted in the United States found that sarcoidosis was the underlying cause of death in 58.8% of deceased sarcoidosis patients. That same study also found that a cardiac etiology (ischemia or myocardial infarction, sudden cardiac death or arrhythmia, congestive heart failure, or cardiomyopathy) contributing to death was present in 24.9% of the 25- to 84-year-old cohort of sarcoidosis patients, and it was also more common in this cohort of patients than in the corresponding age group in the general population [10]. A study that is based on the cohort of sarcoidosis patients presented here found that sarcoidosis was independently associated with heart failure [11].

Most research on the association between sarcoidosis and IHD or related cardiac entities has, to date, been conducted in relatively small cohorts. In their study on 124 sarcoidosis patients, Zöller, Li, Sundquist, and Sundquist found that the standardized incidence ratio (SIR) for coronary heart disease (CHD) was 3.11 in the first year after the first sarcoidosis-associated hospitalization [12]. Another retrospective cohort study that included 345 sarcoidosis patients supported those results, with an estimated 1.58 hazard ratio (HR) for coronary artery disease (CAD) among sarcoidosis patients [13]. In this study, we evaluated the association between sarcoidosis and IHD in a large cohort of patients with sarcoidosis in comparison to a large age- and sex-matched control group.

## 2. Materials and Methods

This retrospective cross-sectional study was conducted using the database of Clalit Health Services (CHS), the largest healthcare organization in Israel, which contains over 4.4 million medical records that were available for analysis. CHS is a health organization that provides state-mandated medical insurance in Israel and operates several university-affiliated hospitals and many clinics throughout the country.

Data on patients with a diagnosis of sarcoidosis were retrieved, and each sarcoidosis patient was age- and sex-matched to a non-sarcoidosis control subject randomly selected from the CHS database. Only exact matches were included, and frequency matching was utilized.

Data retrieved included any diagnosis that was added to patients’ files since the electronic medical records system was introduced into CHS (at around 2000, the exact year varied between institutions) and until 2016. A diagnosis of sarcoidosis or IHD was defined as at least one documented diagnosis by a physician, either in an outpatient clinic or hospital records.

We used chi-square and *t*-tests to evaluate differences between the study and control groups. Variables associated with IHD were identified by a multivariable analysis employing a logistic regression model. Variables related to increased risk of all-cause mortality were assessed by the log-rank test and survival analysis by the Cox proportional hazards method. Statistical analysis was performed using R Statistical Software (version 3.2.2; R Foundation for Statistical Computing, Vienna, Austria).

## 3. Results

**Figure 1:**
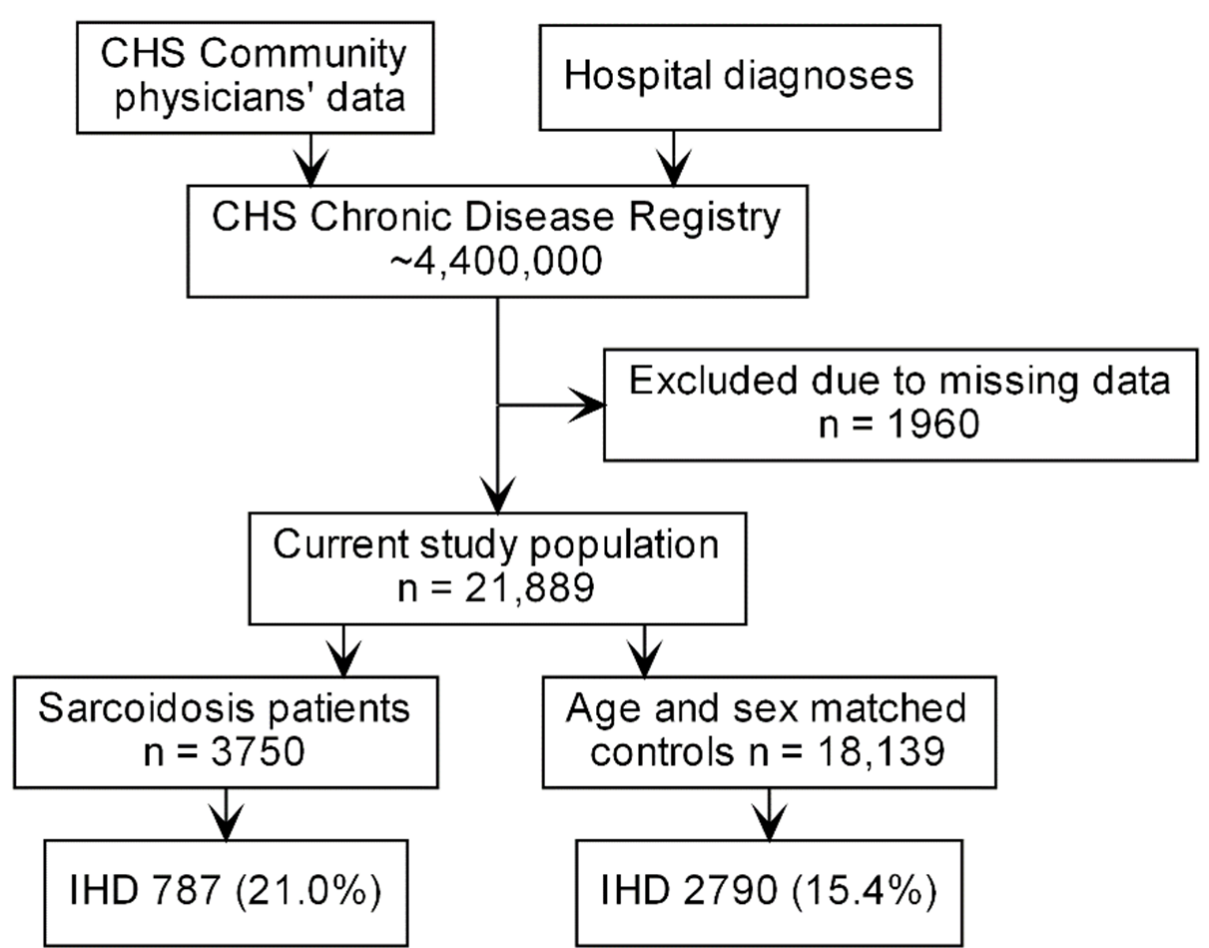
Study flowchart. CHS—Clalit Health Services, N—number of cases, IHD—ischemic heart disease.

The study population included 3750 sarcoidosis patients and 18,139 age- and sex-matched controls (Figure 1).

The characteristics of the study population are presented in Table 1. The sarcoidosis group had a significantly higher risk for systemic hypertension than the control group (51.4% and 44.4%, respectively, *p* < 0.001). A higher proportion of IHD was observed among sarcoidosis patients (21.4%, 856 cases) than among the controls (15.1%, 2999 cases) (*p* < 0.0001).

Table 2 lists the variables associated with IHD. Systemic hypertension, smoking, and a body mass index of over 25 significantly increased the risk for IHD in this cohort. Female gender, however, was inversely associated with IHD risk. An association between sarcoidosis and IHD was demonstrated by a multivariable analysis (adjusted OR 1.5; 95% confidence interval (CI) 1.36–1.66).

Additionally, the sarcoidosis group had a higher rate of mortality: there were 561 (15%) deaths compared with 1636 (9%, *p* < 0.001) deaths for the control group over a period of 15 years. Lastly, Cox proportional hazards methods (Table 3) revealed that sarcoidosis patients had an increased risk for all-cause mortality compared with the controls (adjusted HR 1.93, 95% CI 1.76–2.13).

**Figure 2:**
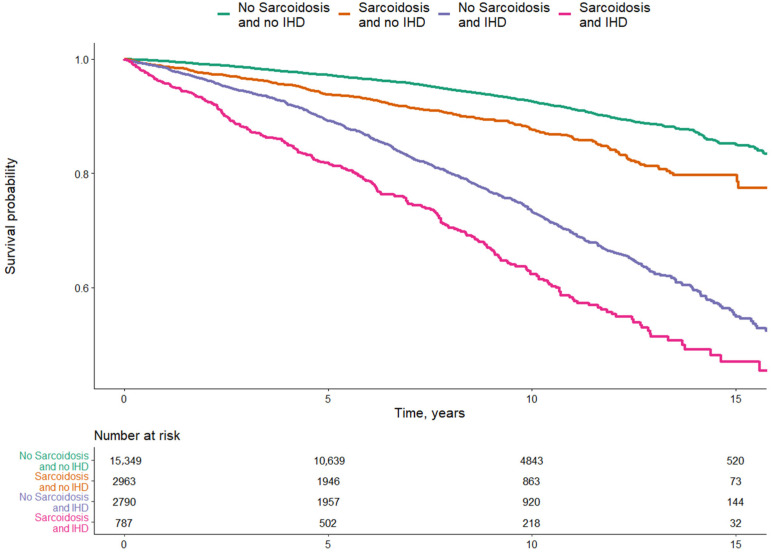
Multivariate analysis of survival probability over a period of 15 years among sarcoidosis patients with and without IHD and controls without sarcoidosis and without IHD.

An increased risk for all-cause mortality was also demonstrated for IHD (adjusted HR 1.66; 95% CI 1.51–1.82). A diagnosis of coexisting IHD and sarcoidosis was associated with the lowest survival probability, while a diagnosis of IHD without sarcoidosis was also associated with reduced survival probability but to a lesser extent. Patients with a diagnosis of sarcoidosis and no IHD had a higher survival probability, which, however, was also reduced compared with the control group (Figure 2).

## 4. Discussion

An increased risk for IHD was reported in many autoimmune diseases, including rheumatoid arthritis, lupus nephritis, inflammatory bowel disease, and Bechet’s disease [14,15,16,17]. Our results show that the prevalence of IHD was, indeed, also increased in sarcoidosis patients, and that mortality among this group was also increased compared with matched controls. Results supporting a higher mortality rate in sarcoidosis patients compared with the general population have appeared in the literature [18,19] and can be attributed to comorbidities (such as those assessed in the current study) as well as to the underlying disease. Our analysis also revealed an increased risk for systemic hypertension in sarcoidosis patients. Mirsaeidi, Omar, Ebrahimi, and Campos [20] observed that systemic hypertension in sarcoidosis patients was associated with higher C-reactive protein levels and an increased erythrocyte sedimentation rate, suggesting a correlation with systemic inflammation.

While cardiac sarcoidosis is a rare but well-described entity that can cause arrhythmias, congestive heart failure, and sudden death [21], only a few studies on the prevalence of IHD or related entities (e.g., coronary heart disease) among sarcoidosis patients have been published to the best of our knowledge. It should be noted, however, that despite its rarity, coronary artery involvement in sarcoidosis has previously been described in the literature [22,23]. A recent systematic review and meta-analysis by Mahmoud et al. [24] (which included a study that is based on this CHS cohort of sarcoidosis patients), found no statistically significant association between sarcoidosis and IHD. It should be noted that the analysis for risk of IHD in sarcoidosis patients included two other studies, one in which the prevalence of acute coronary syndrome was reported in a large cohort of hospitalizations, and another in which the prevalence of myocardial infarction was reported for a smaller cohort. Other studies, however, did find an association between sarcoidosis and entities related to IHD. As previously mentioned, [12] sarcoidosis patients were reported to have an increased SIR (3.11, 95% CI, 2.59–3.71) for CHD in the first year after their first sarcoidosis-related hospitalization. In addition, the risk of CHD for those patients remained elevated for up to five years (SIR 1.41, 95% CI 1.26–1.57) after the index hospitalization, reaching a SIR value of 1.09 after five to ten years. In the aforementioned study it was suggested that the increased risk during the first five years after hospitalization could be caused by increased disease activity leading to atherosclerosis and subsequent CHD.

Another study on cardiovascular disease in sarcoidosis patients was conducted by Ungprasert, Crowson, and Matteson [13], who followed 345 sarcoidosis patients for a period of 15 years. Those patients had a 1.57 HR for cardiovascular disease (CVD) compared with controls. They also had a 1.58 HR for CAD. Our results revealed that sarcoidosis patients have an adjusted OR of 1.57 for IHD, in line with the results of Ungprasert et al. The same group [25] also evaluated the accuracy of the Framingham risk score and the American College of Cardiology/American Heart Association risk score for CVD in sarcoidosis patients. Both risk scores under-evaluated cardiovascular risk among sarcoidosis patients, possibly since neither addressed chronic inflammatory diseases. These results support the need for a different risk stratification approach for sarcoidosis patients.

Some new evidence of sarcoidosis as a risk factor for atherosclerosis and CAD recently emerged. In 2017, Kul et al. conducted a study that included 40 sarcoidosis patients and 42 healthy controls, all without any known traditional risk factors for CAD, in which coronary flow velocity reserve (CFVR) was measured along with other clinical variables [26]. Sarcoidosis was found to be associated with a low CFVR. Interestingly, Kul et al. claimed that disease severity, as well as several clinical variables that reflect disease activity, was not associated with a low CFVR. Their results support those of Ungprasert et al. who showed that immunosuppressive therapy (which could be used as a marker for disease severity) was not associated with a statistically significant increased risk of CVD. With CFVR having been proposed as a biomarker that reflects the severity of CAD [27], these results serve to confirm the notion that sarcoidosis is a risk factor for the development of CAD.

In 2018, a systematic review and meta-analysis of selected studies reported that sarcoidosis was associated with increased arterial stiffness and, therefore, with a higher risk for subclinical atherosclerosis [28]. The authors claimed that despite the fact that atherosclerotic changes generally increase with time, disease duration and sarcoidosis stage did not seem to be associated with worse results in those studies. The occurrence of a pulmonary disease with extrapulmonary manifestations was, however, associated with worse vascular function, which may be explained by an increased inflammatory load causing more severe endothelial dysfunction. In a cross-sectional study that assessed endothelial dysfunction, brachial artery flow-mediated dilation and cell-specific molecule-1 (endocan) levels were used as biomarkers. Sarcoidosis patients who were free of traditional cardiovascular risk factors had increased plasma endocan levels and reduced flow-mediated dilation compared with healthy controls, and a significant inverse correlation was found between those two variables [29].

Several mechanisms through which sarcoidosis could affect the cardiac vasculature and cause IHD have been proposed, apart from direct involvement of the coronary arteries in cardiac sarcoidosis. One likely explanation is that atherosclerosis is associated with systemic inflammation—a notion that is currently widely accepted [30]. The mechanism through which the pro-inflammatory state of sarcoidosis is thought to cause impaired myocardial perfusion involves a reduced activity of endothelial nitric oxide synthase, leading to decreased nitric oxide levels, thereby compromising vasodilation [31]. Another possible mechanism is through disrupted lipid metabolism, which was suggested to play a role in the pathogenesis of sarcoidosis based on findings that sarcoidosis patients with active disease had reduced high-density lipoprotein levels as well as reduced apolipoprotein A1 levels, which can increase atherosclerosis risk [32]. Other possible theories include a shared genetic predisposition that is associated with both sarcoidosis and IHD [13] and a reduction in myocardial perfusion due to pulmonary hypertension and disease that can lead to hypoxemia and microcirculatory anomalies [30].

Our study has several limitations that bear mention. Since this is a retrospective cross-sectional study, the evaluation of the association between sarcoidosis and IHD could be biased by several unidentified confounders. Specific variables that could not be evaluated due to data unavailability were disease severity and disease duration. Information on both of those parameters could have possibly influenced the risk stratification for IHD among sarcoidosis patients and provided more information on that parameter. It should be noted that diagnoses in the CHS database could be made by several types of physicians and in a variety of clinical scenarios. There is, therefore, potential incoherence regarding the diagnosis of IHD in our study, and we were unable to validate the diagnoses due to the size of the study’s groups. Another limitation due to the study’s design is that data regarding the chronological order of IHD and sarcoidosis diagnoses are lacking, and, therefore, we could not conclude which condition preceded the other. Additionally, immunosuppressive and glucocorticoid therapy might also influence IHD risk, given that treatment with immunosuppressive therapy not only indicates disease severity but also possibly acts as an independent risk factor for IHD [33]. The study’s main strength is that its sample size is, to the best of our knowledge, one of the largest to evaluate the association between sarcoidosis and IHD.

## 5. Conclusions

The results of the current study, taken together with those in the relevant literature, serve to support the need to raise the level of awareness of clinicians to the cardiovascular health of sarcoidosis patients. Given the increased mortality in the cohort with coexisting sarcoidosis and IHD that was demonstrated in the results of our study, it is reasonable to argue that treatment and follow-up of sarcoidosis patients should focus not only on the primary disease but also on the possible comorbidity of atherosclerosis and IHD. This calls for managing other risk factors for cardiovascular disease to reduce the subsequent risk of morbidity and mortality among patients with sarcoidosis.

## Figures and Tables

**Table 1:** Characteristics of the study population.

Characteristic	Sarcoidosis (*n* = 3750)	Controls (*n* = 18,139)	*p*-Value
Age, years (mean ± SD)	55.9 ± 14.8	56.3 ± 14.8	0.136
Female gender	2384 (63.6%)	11,630 (64.1%)	0.528
BMI, kg/m^2^ (mean ± SD)	28.7 (6.32)	27.9 (5.78)	<0.001
Socioeconomic status			
Low	1545 (41.7%)	6789 (37.8%)	
Medium	1491 (40.2%)	7336 (40.9%)	0.005
High	673 (18.1%)	3828 (21.3%)	<0.001
Systemic hypertension	1926 (51.4%)	8057 (44.4%)	<0.001
Smoking	1286 (34.3%)	6542 (36.1%)	0.039

Abbreviations: SD—standard deviation, BMI—body mass index.

**Table 2:** Logistic regression for covariates associated with ischemic heart disease.

Characteristic	Odds Ratio	Confidence Interval	*p*-Value
Sarcoidosis	1.5	1.36–1.66	<0.001
Age at diagnosis	1.06	1.05–1.06	<0.001
Female gender	0.39	0.35–0.42	<0.001
Body mass index *			
20–25	1.34	1.008–1.83	0.04
25–30	1.74	1.31–2.35	<0.001
>30	2.12	1.59–2.87	<0.001
Systemic hypertension	4.26	3.84–4.72	<0.001
Smoking	1.6	1.47–1.74	<0.001

* Body mass index of less than 20 was used as reference for this analysis.

**Table 3:** Cox proportional hazards analysis for variables associated with all-cause mortality.

Characteristic	Hazard Ratio	Confidence Interval	*p*-Value
Sarcoidosis	1.93	1.76–2.13	<0.001
Age at diagnosis *	1.1	1.09–1.10	<0.001
Female gender	0.77	0.70–0.85	<0.001
Body mass index **			
20–25	0.51	0.41–0.62	<0.001
25–30	0.43	0.35–0.53	<0.001
>30	0.52	0.43–0.64	<0.001
Systemic hypertension	1.36	1.22–1.52	<0.001
Smoking	1.22	1.11–1.34	<0.001
Ischemic heart disease	1.66	1.51–1.82	<0.001

* Risk per year. ** Body mass index of less than 20 was used as reference for this analysis.

## Data Availability

The datasets for this study cannot be made available due to the nature of personal information they contain.
